# Effects of Essential Oils as Antioxidant and Cryoprotective Agents in Improving Frozen and Thawed Human Sperm Criteria

**DOI:** 10.3390/antiox14010075

**Published:** 2025-01-10

**Authors:** Hamza Goujet, Ismail Kaarouch, Abderrahim Malki, Modou Mamoune Mbaye, Rosalie Cabry, Noureddine Louanjli, Taha Rhouda, Moncef Benkhalifa

**Affiliations:** 1 Laboratory of Physiopathology and Molecular Genetics, Ben M’Sik Faculty of Sciences, Hassan II University of Casablanca, Casablanca 20670, Morocco; abderrahim.malki@univh2c.ma (A.M.); taha.rhouda@univh2c.ma (T.R.); 2 Reproductive Medicine, Reproductive Biology and Genetics, Peritox Laboratory, University Hospital and School of Medicine, Picardie University Jules Verne, 80054 Amiens, France; cabry.rosalie@chu-amiens.fr; 3 Laboratory of Medical Analyses, Reproductive Biology, LABOMAC, IRIFIV Centre, Casablanca 20100, Morocco; mbayeass87@gmail.com (M.M.M.); n.louanjli@gmail.com (N.L.); 4 African Fertility Center, Private Clinic of Human Reproduction and Endoscopic Surgery, Casablanca 20000, Morocco; ismail.fsr@gmail.com

**Keywords:** sperm cryopreservation, essential oils, fertility preservation, sperm quality

## Abstract

Sperm cryopreservation provides patients undergoing oncological, surgical, or infertility treatments the opportunity to conceive their own children, using assisted reproductive technologies. However, the freezing-thawing process can negatively influence both the quantity and the quality of spermatozoa, mainly due to an excessive production of reactive oxygen species and/or an impaired antioxidant defense system in sperm. Aromatic and medicinal plants synthesize essential oils with antioxidant proprieties as a part of their ecological adaptation to environmental stress, thanks to their rich bioactive phytochemical components. This study aimed to assess sperm progressive motility, viability, plasma membrane functionality, and lipid peroxidation levels of human cryopreserved normozoospermic (n = 51) and asthenozoospermic (n = 51) samples without or with the addition of *Thymus satureoides* (TSEO) (20 µg/mL), *Artemisia vulgaris* (AVEO) (48 µg/mL), and *Rosmarinus officinalis* (ROEO) (13 µg/mL) essential oils. Sperm parameters were significantly better preserved with ROEO in both normozoospermic (*p* < 0.05) and asthenozoospermic samples (*p* < 0.01). In contrast, TSEO had a negative impact for both groups (*p* < 0.05). Meanwhile, no significant effects were observed with AVEO. In summary, the study revealed that in vitro addition of essential oils as antioxidant agents during cryopreservation can be either beneficial, which helps preserve sperm parameters and fertilizing potential, or detrimental as spermicidal agents.

## 1. Introduction

Human sperm cryopreservation offers patients undergoing treatments that may cause infertility or sterility, a second chance to conceive through in vitro fertilization technique (IVF) [1,2]. This process temporarily halts the metabolic and functional activity of sperm cells, preserving their fertilization capacity at −196 °C [3]. It relies on cryoprotective agents to shield spermatozoa from the effects of extreme temperatures and prevent ice crystal formation, which could otherwise damage their cellular components [4]. Although spermatozoa are considered unique and less susceptible to damage during the freezing and thawing process due to their high membrane fluidity and low water content [5]. In fact, numerous studies have indicated that cryopreservation adversely affects sperm organelles [6,7], through the oxidation of the plasma membrane’s polyunsaturated fatty acids (PUFAs) and the formation of malondialdehyde (MDA), impairment of mitochondrial function, and the alteration of DNA integrity [8,9]. Additionally, cryopreservation can adversely affect sperm activity by decreasing motility, exacerbating metabolic changes, damaging plasma membrane function, and increasing apoptosis [10,11].

Reactive oxygen species (ROS) are free radicals produced by spermatozoa in low concentrations to support their maturation and physiological functions [12]. However, excessive ROS production can result in oxidative stress, which compromises sperm quality and functionality by damaging motility, viability, plasma membrane integrity, and the structure of genetic material [13,14].

Numerous studies have linked cryodamage at both molecular and functional levels to oxidative stress [15], as spermatozoa are small, have a reduced cytoplasmic volume, and lack protective mechanisms, such as antioxidant enzymes, to counteract the excess of ROS generated during the freezing and thawing processes [16].

Moreover, osmotic stress can arise during the freezing process, when a cryoprotective agent is added, resulting in an increased concentration of extracellular solutes in a hypertonic environment, which leads to the dehydration of spermatozoa to maintain an equilibrium (homeostasis) based on the phenomenon of osmosis. During thawing, this situation is reversed as spermatozoa are exposed to a hypotonic medium, which leads them to swell after penetrating the water. The latter could be more dangerous for sperm integrity as the water penetrating the cell could be rich in reactive oxygen species [17,18]. Some studies have shown that the addition of an antioxidant agent to sperm during cryopreservation can help to eliminate the excess ROS and minimize their effects on the post-thaw quality and functionality of sperm of a certain species [19,20,21]. In addition, a balanced diet based on lipids and n-3 fatty acids of plant origin and some nutrients could improve sperm properties and make them more resistant to damage caused by cryopreservation [22,23,24].

Essential oils are secondary metabolites of medicinal and aromatic plants, characterized by their composition rich in phytochemicals with a diversity of pharmacological activities, like the antioxidant power, and they are synthesized to ensure some essential activities in their ecosystems [25,26].

In the field of reproductive biology, the phytochemical compounds of certain essential oils have been used in specific doses to enhance sperm quality parameters, such as thyme and rosemary, which belong to the Lamiaceae family and contain a variety of biomolecules such as thymol, carvacrol, p-cymene, γ-terpinene, linalool, carnosol, and carnosic acid [27,28,29]. Also, mugwort, which is a subtype of the Asteraceae family with a variety of components such as camphor, camphene, caryophyllene oxide, α and β-thujone [30]. In addition, numerous studies have evaluated the effects of adding them in vivo or in vitro at a specific concentration with the aim of exploiting their antioxidant power to enhance the motility, vitality, and concentration of spermatozoa, with a major action on stabilizing the oxidant/antioxidant balance, and they demonstrated some interesting results [31,32,33,34].

Furthermore, essential oils from specific plants, including *Rosmarinus officinalis*, *Thymus satureoides*, and *Artemisia vulgaris*, may serve as alternatives to help regulate ROS levels due to their rich antioxidant biomolecules, which is worth investigating the effects of their inclusion during sperm cryopreservation as a promising solution to reduce the impact of oxidative stress on sperm quality. However, their use must be approached with caution, as the effects depend mainly on the composition and concentration used of each essential oil, and in some cases, they can have a negative impact on the components of spermatozoa, such as the plasma membrane and DNA integrity [35]. This study aimed to assess the effects of supplementing cryopreserved human sperm samples with three essential oils recognized for their antioxidant properties: *Thymus satureoides*, *Rosmarinus officinalis*, and *Artemisia vulgaris*. The evaluation focused on their impact on progressive motility, viability, plasma membrane integrity, and lipid peroxidation levels.

## 2. Materials and Methods

### 2.1. Plant Materials

The essential oils of interest evaluated in this study were obtained from three different plant species: *Thymus satureoides*, *Rosmarinus officinalis*, and *Artemisia vulgaris*, collected randomly from different mountain regions of Morocco. Bearing in mind that the concentration of bioactive components depends mainly on the stage and time of harvesting, as well as on washing and drying techniques [36], plants are harvested at the right time of year, gently rinsed in fresh water, and dried in the shade for a sufficiently long period [37,38] (Table 1).

### 2.2. Essential Oil Extraction

Essential oils were extracted using the Clevenger hydrodistillation technique, which is the conventional method for preserving phytochemical compounds of interest [36]. Distilled water and plant material were placed in a heated 2 L flask, combined with a refrigeration system to ensure condensation of the essential oil for a sufficiently long period. The distillation process ends with an aqueous (aromatic water) and an organic (essential oil) phases. The latter was collected in a sealed amber vial and stored at 4 °C to preserve the biomolecules of interest.

The yield (Y) of each essential oil was determined using the following formula:

$$Y=\frac{v}{w}\times 100$$

v: The volume of the collected essential oil. w: Plant material weight.

### 2.3. Study Design

The effects of in vitro supplementation with the essential oils of TSEO, ROEO, and AVEO on human cryopreserved sperm parameters were analyzed in an experimental study. Semen samples were collected from the Laboratory of Medical Analysis and Reproductive Biology (LABOMAC) and the In Vitro Fertilization Centre (IRIFIV) of the IRIS Clinic, in which the consent of each patient was obtained before the use of the samples following a full explanation of the aim, methods, and how the results of the study in question would be exploited. Afterward, the assessment of spermatozoa progressive motility, lipid peroxidation levels, plasma membrane functionality, and viability were evaluated at the Laboratory of Physiopathology and Molecular Genetics (LPGM) of the Ben M’sik Faculty of Science, Casablanca, Morocco.

### 2.4. Participants

The semen samples used in this study were obtained from 102 volunteer male patients aged between 18 and 50, who planned an infertility evaluation. For that purpose, and according to World Health Organization (WHO) standards [39], semen samples classified with normozoospermia (progressive motility ≥ 30%) (n = 51) or asthenozoospermia (progressive motility < 30%) (n = 51), with a concentration ≥ 16 million sperm/mL, viability ≥ 54%, and 2–6 days of sexual abstinence, were included. However, abnormal semen samples that were received from patients with ejaculation difficulties, a random or habitual exposure to a toxic environment, and/or a high duration of sexual abstinence were excluded.

### 2.5. Sperm Collection Procedures

Semen samples are collected by masturbation in sterile and labeled containers. In addition, after they were liquefied (30–60 min/35 °C), an assessment of sperm quantitative and qualitative parameters was performed using IVOS CASA automated system software, version V14.0. Hamilton Thorne (Beverly, MA, USA), to ensure that they are categorized as normozoospermic or asthenozoospermic.

### 2.6. Essential Oils Treatment

In order to determine the optimum concentration of the essential oils of interest for sperm cryopreservation, without adversely affecting their motility and viability, serial dilution was carried out using 0.5% DMSO, as the essential oils are insoluble in water [40]. With a final volume of 1 mL (200 µL of the essential oil and 800 µL of the diluent) for each tube. Sperm samples (n = 20) were then pre-treated using a density gradient technique to remove seminal plasma and adjust the concentration to 10 million sperm/mL. After centrifugation (1200× *g* for 20 min), the resultant pellet was fractionated into a control and a supplemented group, followed by an incubation with each concentration of essential oil at 35 °C and 5% CO_2_. After 4 h of incubation [41], cell motility was measured using IVOS CASA automated system software, version V14.0. Hamilton Thorne (Beverly, MA, USA). While viability was assessed by eosin-nigrosine staining. The ideal concentration was selected depending on the rate of motile and viable cells.

### 2.7. Pre-Processing of Semen Samples

The collected semen samples were pre-processed by the density gradient technique employing Puresperm gradients (Gynemed, Rostock, Germany), by inserting 1 mL PureSperm 80%, 1 mL PureSperm 40%, and 1 mL of the semen sample into a 15 mL Falcon tube, respectively. After centrifugation (1200× *g* for 20 min), the resulting pellet was kept for cryopreservation. To obtain a uniform sperm concentration and to ensure that the observed effect was caused uniquely by the essential oils of interest, the pellet of each sample was diluted with Earle′s Balanced Salt Solution (EBBS) to achieve a final concentration of 10 million sperm/mL. In addition, SpermFreeze (Fertipro, Beernem, Belgium) was added as a cryostorage medium at an equal volume. It is composed of glycerol, physiological salts, glycine, glucose, and lactate. The final solution was divided into 4 straws: control group (G0), group supplemented with either ROEO (13 µg/mL) (G1) or TSEO (20 µg/mL) (G2) or AVEO (48 µg/mL) (G3). Moreover, the prepared straws were subjected to a slow freezing program starting from 24 °C to −10 °C with a cooling rate of 0.5 °C/min, progressing from −10 °C to −60 °C (−5 °C/min) before being plunged into liquid nitrogen to reach a final temperature of −196 °C. Finally, they were stored in a stable container until the day of thawing.

### 2.8. Assessment of TSEO, ROEO, and AVEO Effects on Spermatozoa Motility, Viability, Lipid Peroxidation Levels, and Plasma Membrane Functionality

To assess the impact of *Rosmarinus officinalis*, *Thymus satureoides*, and *Artemisia vulgaris* essential oils on human sperm criteria before and after cryopreservation, the progressive motility, viability, lipid peroxidation levels, and plasma membrane functionality were evaluated in both normozoospermic and asthenozoospermic semen samples.

#### 2.8.1. Human Cryopreserved Sperm Motility Evaluation

The progressive motility of human cryopreserved spermatozoa was evaluated with or without the addition of *Thymus satureoides*, *Rosmarinus officinalis*, and *Artemisia vulgaris* essential oils. For this purpose, cryopreserved straws were thawed by placing them in a calibrated incubator at a temperature of 37 °C and 5% of CO_2_ for 1 min and then centrifuged (200× *g* for 10 min) to remove the cryoprotective agent. A total of 20 µL of the thawed sperm sample from each group was placed in the Makler counting chamber of 10 µm to visualize sperm motility using IVOS CASA automated system software, version V14.0. Hamilton Thorne (Beverly, MA, USA).

#### 2.8.2. Human Cryopreserved Sperm Viability Assessment

Sperm cell viability has been analyzed with the one-step eosin-nigrosine staining technique, which is dependent on the nature of the spermatozoa’s plasma membranes. In this method, only dead sperm cells are stained pink, while live ones remain colorless [42,43]. For this purpose, 20 µL of semen sample is mixed with a drop of eosin-nigrosine under a coverslip and slide with the aid of a counter, 100 spermatozoa are counted under a white-light microscope with a magnification of 40×.

#### 2.8.3. Plasma Membrane Functionality

The functionality of the cryopreserved sperm plasma membrane and its ability to fertilize were assessed by the hypo-osmotic swelling test (HOST), by examining its response when exposed to a hypoosmotic solution inducing or not a swelling of the sperm tail. To do this, in accordance with the manufacturer’s instructions, 0.3 mL of thawed semen sample from each group is combined with 1 mL of HOS solution, then gently mixed and incubated for 1 h at 35 °C and 5% CO_2_. A drop of the mixture is placed between a slide and a coverslip. Then, using a counter, 200 spermatozoa are observed under a phase-contrast microscope at 40-× magnification.

#### 2.8.4. Lipid Peroxidation Assessment

The level of lipid peroxidation in each cryopreserved sperm sample was assessed by the Thiobarbituric Acid Reactive Substances Test (TBARS), which measures the concentration of malondialdehyde (MDA), a metabolite formed by the action of free radicals (ROS) on polyunsaturated fatty acids of the sperm plasma membrane. The concept of this test is based on the ability of thiobarbituric acid (TBA) to create a colorimetric conjugate with MDA molecules, which is proportional to its concentration and can be measured using a spectrophotometer. For this, 0.25 mL of the resulting pellet after centrifugation is added to 0.3 mL trichloroacetic acid (TCA) and 0.5 mL thiobarbituric acid (TBA) in a glass tube. The tubes are heated at 95 °C for 1 h, cooled for 10 min, and centrifuged at 300× *g*/10 min. The absorbance of the supernatant is measured on a spectrophotometer at 532 nm, with a molar absorption coefficient of 1.56 × 10^5^ mole/L/cm.

### 2.9. Statistical Analysis

The obtained results in this study were analyzed statistically using SPSS statistics software 27.0.1. The Shapiro–Wilk test was used to assess the normality of each group, and the Box–Cox transformation was used to respect the requirements of parametric analysis. The ANOVA test with repeated measures has been used to investigate the impact of cryopreservation on human sperm without (G0) or with the supplementation of the essential oils of interest (G1, G2, and G3). Post hoc pairwise comparisons using the Bonferroni correction were conducted in order to determine whether there were significant differences between treatment groups. All data are represented as mean ± standard error of the mean (SEM), and a *p*-value < 0.05 was considered statistically significant.

## 3. Results

### 3.1. The Effect of Sperm Incubation with Different Essential Oil Concentrations

After incubating sperm cells with the essential oils at different concentrations over a period of 4 h, the most appropriate dose was chosen depending on the percentage of motility and viability as follows: for ROEO 13 µg/mL (1/125), for TSEO 20 µg/mL (1/625), and for AVEO 48 µg/mL (1/625). However, other concentrations (e.g., 1/5, 1/25) were excluded since their addition resulted in a lower percentage of each parameter studied compared to the control group (G0) (Table 2).

### 3.2. The Essential Oils Effects on the Progressive Motility

Sperm progressive motility has decreased significantly after cryopreservation compared with fresh sperm samples (*p* < 0.001). Moreover, it was lower for group G2 (TSEO) on normozoospermic and asthenozoospermic semen samples (*p* = 0.006). However, a higher percentage of progressive motility was observed in-group G1 (ROEO), in which it was higher in the normozoospermic samples (*p* = 0.039) than the asthenozoospermic (*p* = 0.045). Meanwhile, no significant difference was observed for group G3 (AVEO) (*p* > 0.05) (Table 3).

### 3.3. The Essential Oils Effects on the Viability

The viability of human spermatozoa diminished significantly after the process of freezing/thawing compared to the fresh sperm samples (*p* < 0.001). In addition, a significant elevation was observed in G1 asthenozoospermic samples compared to normozoospermic samples, for which it was slightly higher (*p* = 0.003). Furthermore, group G2 showed a significant reduction in viability percentage for both samples with normozoospermia (*p* = 0.017) and asthenozoospermia (*p* = 0.008). However, group G3 showed an increase in the number of viable spermatozoa compared to the control group (G0), but this increase was not statistically significant (*p* > 0.05) (Table 3).

### 3.4. Essential Oils Effects on Spermatozoa Plasma Membrane Functionality

The obtained results demonstrated that the percentage of live spermatozoa with swollen tails decreased significantly after cryopreservation for samples with normozoospermia (*p* = 0.011) and asthenozoospermia (*p* = 0.023). The addition of ROEO for group G1 resulted in a significant increase in the proportion of HOS-positive for normozoospermic (*p* = 0.015) and asthenozoospermic samples (*p* = 0.002) compared to the control group (G0). In-group G2, TSEO addition caused a higher decrease in the percentage of HOS-positive samples for samples with normozoospermia (*p* = 0.007) compared with asthenozoospermia (*p* = 0.325). Furthermore, AVEO (G3) did not show any significant differences (Table 3).

### 3.5. Lipid Peroxidation Level

TBARS assay revealed an increase in MDA levels after cryopreservation for both normozoospermic (*p* = 0.002) and asthenozoospermic (*p* = 0.006) semen samples. The level of MDA was low for group G1 compared with group G0, in which it was slightly lower in samples with normozoospermia (*p* = 0.016) than asthenozoospermia (*p* = 0.002). For G2 and G3 groups, no significant differences were observed (*p* > 0.05) (Table 3).

## 4. Discussion

Sperm banking maintains spermatozoa functionality and quality by temporarily stopping its metabolic activities for further use [8]. However, many studies have mentioned that spermatozoa cryopreservation could negatively affect its fertilizing ability due to the action of low temperatures, oxidative and osmotic stress after the process of freezing and thawing [44,45,46]. Due to this problem, many studies have been focused on finding innovative additives and nutrients as solutions to improve the procedures of cryopreservation and preserve the properties of cryopreserved spermatozoa [7,47,48,49].

In this study, we compared the impact of cryopreservation on the progressive motility, viability, plasma membrane functionality, lipid peroxidation levels of human normozoospermic and asthenozoospermic semen samples, followed by the assessment of the action of *Rosmarinus officinalis* (13 µg/mL), *Artemisia vulgaris* (20 µg/mL), and *Thymus satureoides* (48 µg/mL) essential oils during cryopreservation, as antioxidant and cryoprotective agents. In results, we found that human cryopreserved sperm quality could be deteriorated after the process of freezing/thawing, by significant changes in the studied parameters, and those variations are more important in the asthenozoospermic group than the normozoospermic ones.

As far as we know, this is the first study to find that the addition of certain essential oils known for their antioxidant potency to human sperm during cryopreservation could either be beneficial by enhancing its functionality and preserving its fertilizing ability or, on the contrary, damaging it as a spermicidal agent.

Freezing and thawing processes induced a significant impairment of human sperm criteria compared to fresh samples, and these findings were more evident for asthenozoospermic than normozoospermic sperm samples. These differences could be directly attributed to the nature of asthenozoospermic spermatozoa, which are more vulnerable to the action of oxidative stress as they have mitochondrial dysfunction responsible for energy and motility deficiency, high concentrations of MDA molecules, as well as the presence of immature and abnormal spermatozoa that produce ROS in high amounts [50,51,52,53].

Interestingly, a significant improvement of human cryopreserved sperm characteristic parameters was observed after thawing in group G1 supplemented with ROEO (13 µg/mL) compared to the control group (G0) by an increase in spermatozoa progressive motility, viability, and plasma membrane functionality, and a decrease in lipid peroxidation levels for both normozoospermic and asthenozoospermic semen samples.

The obtained results could be explained by the antioxidant activity of ROEO, which is associated with its phytochemical composition that contains a diversity of molecules such as 1,8-Cineole, Camphene, α-Pinene, Camphor, and α-Thujene [54,55], which are known to have an antioxidant power. We think that its action could be either performed by supporting the intracellular antioxidant system of spermatozoa and/or by scavenging the excess of ROS free radicals and minimizing their effects.

Those results are in accordance with the study conducted by Touazi et al. [56], which investigated the effect of *Rosmarinus officinalis* essential oil on the motility and kinetic parameters (VSL, VCL, VAP, ALH, and BCF) of rooster semen at 0, 6, 24, and 48 h of short-term storage at 4 °C. They found that the concentration of 8.7 µg/mL had a positive effect, especially after 6 h storage. However, the concentration of 870 µg/mL showed a negative impact by a spermicidal effect after 24 h of storage. In addition, the study led by Malo et al. [57] had evaluated the effect of adding rosemary extract as an antioxidant agent during the cryopreservation of boar epididymal spermatozoa. They discovered an improvement over the control group of sperm motility, viability, and plasma membrane integrity at a concentration of 10 g/100 mL after 2 h of incubation without affecting the state of the acrosome, and low MDA levels. In a similar study, Motlagh et al. [58] evaluated the effect of adding the aqueous extract of *Rosmarinus officinalis* on thawed ram spermatozoa’s motility, velocity parameters, plasma membrane functionality, vitality, capacitation, acrosome status, and lipid peroxidation levels. The authors observed that there was an effect, which depended on the concentration of aqueous extract, in which sperm quality is preserved at low concentrations, yet high concentrations have a negative impact.

Adverse effects were noted in the *Thymus satureoides* essential oil-supplemented G2 group (20 µg/mL) compared to the control group (G0) for both normozoospermic and asthenozoospermic sperm samples. This finding may be attributable to the two major phytochemical compounds that are common to all thyme species: thymol and carvacrol, which are known to be powerful antioxidants but also to have anti-proliferative activity, in which they can induce cell apoptosis, damage of plasma and mitochondrial membrane proteins, morphological changes, and cytoplasmic leakage [59,60,61].

According to the study of Kchikich et al. [62], which evaluated the impact of *Thymus satureoides* essential oil on the quality of ben Arouss buck semen during storage at a temperature of 4 °C. They demonstrated that its effect depends essentially on the dose used, where a concentration of 0.05% is detrimental to sperm parameters, while on the contrary, a concentration of 0.01% is beneficial and significantly improves the quality of thawed semen. Similarly, the study by Aya et al. [63] showed that the addition of thyme extract nanoformulation to cryopreserved goat sperm significantly improves the progressive motility, vitality, and plasma membrane integrity. They also noted that it could reduce sperm apoptosis, MDA levels, and chromatin decondensation, and increase antioxidant capacity and catalase activity. The divergences between their findings and ours might be explained by the species of sperm samples, the nature of the thyme extract, and the cryopreservation procedure followed.

Bhandari et al. [64] found that incubation of mouse spermatozoa with the ethanolic extract of *Artemisia vulgaris* could provoke an early acrosomal reaction and a loss of viability, but with only minimal damage to the membrane. Furthermore, they also reported that the ethanolic extract could block fertilization by inducing an early acrosomal exocytosis and could be considered as a potential contraceptive after an intravaginal administration. Concerning our study, the addition of *Artemisia vulgaris* essential oil in the G3 group showed no statistically significant differences when compared with the control group (G0) for either normozoospermic or asthenozoospermic sperm. This may be primarily associated with the concentration used in this study, which might be too low to demonstrate any significant effects, and to its low yield.

With this in mind, human sperm preservation procedures could be improved by adding *Rosmarinus officinalis* essential oil as an antioxidant additive, as it would minimize the negative impact of freezing and thawing on sperm activity and quality. On the other hand, *Thymus satureoides* could be used as a spermicidal agent.

For the limitations of the current study, other extracts should be studied to compare the obtained results and ensure any observed effects occur only after the addition of the extract of interest as an antioxidant agent. It would also be necessary to study its effects on sperm samples from testicular biopsies and assess other sperm parameters, such as the capacitation status, acrosome integrity, and fertilizing ability. In addition, it would also be necessary to determine at the molecular level the phytochemical compounds responsible for the observed results.

## 5. Conclusions

The results of this study may provide evidence for the incrimination of oxidative stress in the etiology of impaired cryopreserved sperm parameters. Also, it supports the idea of using essential oils as an antioxidant agent by reducing the level of MDA, which is a marker of lipid peroxidation resulting from the action of free radicals on plasma membrane polyunsaturated fatty acids, as well as preserving sperm cells progressive motility, viability, and plasma membrane functionality.

## Figures and Tables

**Table 1:** Properties of plants used for extracting the essential oils.

Plants Scientific Names	Plant Family	Plant Parts	Harvesting and Drying	Location	Weight (g)	Quantity of Distilled Water [mL]	Yield (%)
*Thymus satureoides*	Lamiaceae	Leaves	July (4 days of drying at 20 °C)	Agadir (Anti-atlas)	100	1000	2.4%
*Rosmarinus officinalis*	Lamiaceae	Leaves	May (3 days of drying at 27 °C)	Taourirt (Eastern rif)	100	1000	2.5%
*Artemisia vulgaris*	Asteraceae	Leaves	April (5 days of drying at 27 °C)	Taza (Middle-atlas)	250	1200	1%

**Table 2:** Percentage of sperm progressive motility and viability before and after essential oils supplementation (n = 20).

Sperm Parameters	ROEO	TSEO	AVEO
**Motility** (For G0 = 75%) **^1^**	[0] = 0% **^2^**	[0] = 0% **^2^**	[0] = 0% **^2^**
	[1/5] = 11%	[1/5] = 5%	[1/5] = 9%
	[1/25] = 28% [1/125] = 77% [1/625] = 66%	[1/25] = 8% [1/125] = 52% [1/625] = 76%	[1/25] = 35% [1/125] = 58% [1/625] = 58% [1/3125] = 74%
**Viability** (For G0 = 85%) **^1^**	[0] = 0% **^2^**	[0] = 0% **^2^**	[0] = 0% **^2^**
	[1/5] = 14% [1/25] = 27% [1/125] = 83% [1/625] = 75%	[1/5] = 8% [1/25] = 61% [1/125] = 89% [1/625] = 86%	[1/5] = 25% [1/25] = 66% [1/125] = 78% [1/625] = 82%

**^1^** The values of sperm progressive motility and viability without the supplementation of the essential oils. **^2^** The effect of adding the essential oil directly without dilution.

**Table 3:** Human sperm progressive motility, viability, plasma membrane functionality (HOST) and lipid peroxidation levels (MDA) before and after cryopreservation in normozoospermia and asthenozoospermia samples, without (G0) or with the addition of *Rosmarinus officinalis* (G1), *Thymus satureoides* (G2), and *Artemisia vulgaris* (G3) essential oils. All data are expressed as mean ± mean standard error (SEM). The statistical analysis was conducted employing ANOVA with Bonferroni correction, where *p* < 0.05 was considered significant.

Sperm Categories	Groups	Progressive Motility (%)	Viability (%)	HOST (%)	MDA (nmol/L)
Normozoospermia	A	43.80 ± 1.153	57.08 ± 0.238	39.71 ± 1.382	0.972 ± 0.04129
	G0	23.79 ± 1.828	29.39 ± 2.070	34.16 ± 1.486	1.130 ± 0.068
	G1	30.98 ± 1.985 ^1^	37.49 ± 2.133 **^2^**	40.88 ± 1.570 **^1^**	0.870 ± 0.068 **^1^**
	G2	15.71 ± 1.272 ^2^	21.24 ± 1.380 **^2^**	26.43 ± 1.898 **^2^**	1.087 ± 0.0769
	G3	23.55 ± 1.648	23.80 ± 2.097	35.27 ± 1.403	1.068 ± 0.074
Asthenozoospermia	A	22.11 ± 0.739	28.88 ± 0.727	32.44 ± 1.750	1.186 ± 0.0758
	G0	18.65 ± 0.875	22.94 ± 1.497	29.40 ± 1.943	1.573 ± 0.109
	G1	22.18 ± 0.859 ^1^	32.43 ± 1.631 **^2^**	38.92 ± 1.931 **^2^**	1.013 ± 0.088 **^2^**
	G2	14.43 ± 1.091 ^2^	18.53 ± 1.221 **^1^**	25.47 ± 1.747	1.201 ± 0.092
	G3	21.22 ± 0.798	20.57 ± 1.471	30.21 ± 2.168	1.541 ± 0.097

A: Before cryopreservation; ^1^ Statistically significant with a *p* < 0.05; ^2^ Statistically significant with a *p* < 0.01.

## Data Availability

All the data included in this study can be obtained on request.
